# Evaluation of scrub typhus diagnosis in China: analysis of nationwide surveillance data from 2006 to 2016

**DOI:** 10.1186/s40249-019-0566-0

**Published:** 2019-06-29

**Authors:** Hua-Lei Xin, Jian-Xing Yu, Mao-Gui Hu, Fa-Chun Jiang, Xiao-Jing Li, Li-Ping Wang, Ji-Lei Huang, Jin-Feng Wang, Jun-Ling Sun, Zhong-Jie Li

**Affiliations:** 1 Qingdao City Center for Disease Control and Prevention, Qingdao, 266033 Shandong China; 2 Key Laboratory of Surveillance and Early Warning on Infectious Disease, Division of Infectious Disease, Chinese Center for Disease Control and Prevention, Beijing, 102206 China; 3 Ministry of Health Key Laboratory of Systems Biology of Pathogens and Dr. Christophe Mérieux Laboratory, CAMS-Fondation Mérieux, Institute of Pathogen Biology, Academy of Medical Sciences of China and Peking Union Medical College, Beijing, 100000 China; 4 State Key Laboratory of Resources and Environmental Information System, Institute of Geographic Sciences and Natural Resources Research, Chinese Academy of Sciences, Beijing, 100000 China; 5 National Institute of Parasitic Diseases, Chinese Center for Disease Control and Prevention, Shanghai, 200025 China

**Keywords:** Scrub typhus, Diagnosis, Evaluation, China

## Abstract

**Background:**

Scrub typhus is a life-threatening disease caused by *Orientia tsutsugamushi*, and specific antimicrobial medicine is available*.* Early and accurate diagnosis is essential for reducing the risk of severe complications and death. In this study, we aimed to evaluate the case diagnosis situation among medical care institutions and geographical regions in China, and the results will benefit both clinical practice and the disease surveillance system.

**Methods:**

We extracted individual scrub typhus case data 2006–2016 from a national disease surveillance system in China. The diagnosis category and interval time from illness onset to diagnosis were compared among three levels of medical care institutions and provinces. The descriptive analysis method was performed in our study.

**Results:**

During the 11-year study period, 93 481 scrub typhus cases, including 57 deaths, were recorded in the nationwide surveillance system. The overall proportion of laboratory-confirmed cases was only 4.7%, and this proportion varied greatly among primary medical centres (2.8%), county level hospitals (4.2%), and city level hospitals (6.3%). Notably, the proportion of laboratory-confirmed cases has consistently decreased from 16.3% in 2006 to 2.6% in 2016, and the same decreasing trend was found among all three levels of medical care institutions. The interval from illness onset to case diagnosis (T_diag_) for all cases was 5 days (interquartile range [IQR]: 2–9 days) and decreased from 7 days (IQR: 3–11 days) in 2006 to 5 days (IQR: 2–8 days) in 2016. The risk of death for patients with a T_diag_ of > 7 days was 2.2 times higher (*OR* = 2.21, 95% *CI*: 1.05–5.21) than that of patients with a T_diag_ of < 2 days.

**Conclusions:**

The interval time from illness onset to diagnosis for scrub typhus cases decreased greatly in China; however, the diagnosis rate of cases with laboratory-confirmed results must be increased among all levels of medical care institutions to reduce both the risk of death and the misuse of antibiotics associated with scrub typhus.

**Electronic supplementary material:**

The online version of this article (10.1186/s40249-019-0566-0) contains supplementary material, which is available to authorized users.

## Multilingual abstracts

Please see Additional file 1 for translations of the abstract into the five official working languages of the United Nations.

## Background

Scrub typhus, also called tsutsugamushi disease, is a vector-borne infectious disease primarily endemic to a region called the Asian-Pacific “tsutsugamushi triangle” (South Asia, Southeast Asia, East Asia, the Pacific Islands and Northern Australia) [1, 2], with occasional case reports from Africa, the Middle East, and South America [2, 3]. Accurate incidence data would most likely increase the current estimates of more than two billion people living in scrub typhus-endemic areas and the occurrence of one million cases of the disease annually [3–5]. Furthermore, the incidence in all known endemic regions has begun to rise over the last decade [6–8].

Infection with *Orientia tsutsugamushi* (an obligate intracytosolic bacterium), the causal agent of scrub typhus, can range from mild (asymptomatic) to lethal. The illness is generally flu-like (fever, headache, myalgia) in its symptomology [9] and classically begins with the appearance of an eschar at the site of mite feeding and enlargement of draining lymph nodes, followed by fever, headache, myalgia, and gastrointestinal symptoms. In severe cases, the illness can progress to the development of interstitial pneumonia, acute respiratory distress syndrome, meningoencephalitis, acute kidney injury, or disseminated intravascular coagulation. The mortality rate varies and can be as high 50% unless patients are treated sufficiently early in the course of illness with liposoluble antibiotics such as doxycycline, tetracycline, azithromycin, or somewhat less-effective chloramphenicol [3, 10, 11]. The presence of eschars is considered pathognomonic. However, it has been reported that eschar incidence varies from 7 to 97% in endemic areas, and scrub typhus cases without eschar are clinically indistinguishable from other diseases, including malaria, dengue fever, leptospirosis, other rickettsioses, meningococcal disease, typhoid fever, infectious mononucleosis and HIV [3, 5, 12–14]. Notably, the spotted fever group of rickettsioses, insect bites (including spider bites), posttraumatic scabs and anthrax can include an inoculation eschar at the surface of the skin [3, 9]. All of the above conditions associated with an eschar make the clinical diagnosis of scrub typhus quite difficult and increase the chance of misdiagnosis, underdiagnosis and drug misuse.

In the 1950s, the Chinese government established a routine reporting system for selected infectious diseases, with data available for 31 provinces in China, covering a population of approximately 1.3 billion [15]. This system has been web-based since 2003, and it operates through administrative grading responsibility and territorial management [16]. Thirty-nine notifiable infectious diseases, which are divided into three classes (A, B, and C), are included in the reporting system [17, 18]. Clinicians complete a standard case report card for infectious diseases. Epidemic reports are time-sensitive: all class A infectious diseases, along with the class B infectious diseases of pulmonary anthrax and severe acute respiratory syndrome (SARS), should be reported through the network within 2 h of diagnosis; other class B and all class C infectious diseases should be reported within 24 h [18, 19]. In 2006, scrub typhus was added to the national infectious disease surveillance system as a voluntarily reportable disease, which follows the requirement for class B and C infectious diseases and must be reported within 24 h after diagnosis [20].

Scrub typhus remains a serious public health problem in China, and studies have shown that the annual incidence of scrub typhus has increased since 2006 [14, 21, 22]. Because scrub typhus is rapidly curable by specific antibiotics, early and accurate diagnosis of the disease is essential to reduce the risk of severe complications and death. To understand the diagnostic situation of scrub typhus in China, we examined the current interval time from illness onset to diagnosis and the accuracy of scrub typhus diagnosis. We compared these measures among three levels of medical care institutions by analysing nationwide surveillance data for scrub typhus cases from 2006 to 2016, aiming to evaluate the diagnosability of scrub typhus in medical care institutions in China.

## Methods

### Data source and ethical considerations

In this study, all data for scrub typhus cases from 2006 to 2016 were extracted from the national database of infectious disease of the Chinese Center for Disease Control and Prevention (China CDC). Individual case information for each scrub typhus patient was reported by clinicians to the web-based National Notifiable Infectious Disease Reporting Information System (NNIDRIS) at the China CDC. The information reported by clinicians included demographic information, illness onset date, diagnosis type, reporting date and reporting institution (see Additional file 2).

All data used in this study were anonymous, such that the individual patients could not be identified. Because the national surveillance of scrub typhus was a part of routine public health investigation, the study was exempted from the Institutional Review Board assessment.

### Case definition

In China, scrub typhus cases have been classified as suspected, probable or confirmed [20]. Definition of each term was shown in Table 1.

**Table 1:** Case definition for scrub typhus in China

Term	Definition
Suspected cases	(1) the individual participated in outdoor activities with possible exposure risk, i.e., farming, fishing, camping, and straw collection, during the disease epidemic season (from May to November south of the Yangtze River and from October to November north of the Yangtze River in China) three weeks before illness onset and presented with fever, lymphadenectasis and skin rash, and diagnosis of other common diseases such as typhus fever, dengue fever or epidemic hemorrhagic fever was excluded; (2) or the individual had an uncertain exposure history for mite bites but developed fever, lymphadenectasis and skin rash during the local epidemic season of scrub typhus.
Probable cases	(1) the patient is suspected of having the specific eschars/ulcers of scrub typhus; (2) or the individual participated in an outdoor activity three weeks before illness onset and presented with fever and the specific eschars/ulcers of scrub typhus.
Confirmed cases	(1) a probable case with any of the following four laboratory test results—A) an agglutination titer of ≥1:160 in the Weil-Felix test using the *Proteus mirabilis* OXK strain; B) a fourfold or greater rise in serum IgG antibody titer (diluted from 1:32 in twofold increments) for mixed antigenic slides (including Karp, Kato, Gilliam and Kawasaki) between acute and convalescent sera, as detected using an indirect immunofluorescence antibody assay (IFA) [11, 13]; C) polymerase chain reaction (PCR) detection of the O. tsutsugamushi 56-kDa gene in clinical specimens; or D) isolation of O. tsutsugamushi from clinical specimens; (2) or a suspected case with any of the last three abovementioned laboratory test results (B, C or D).

### Data analysis

**Fig. 1:**
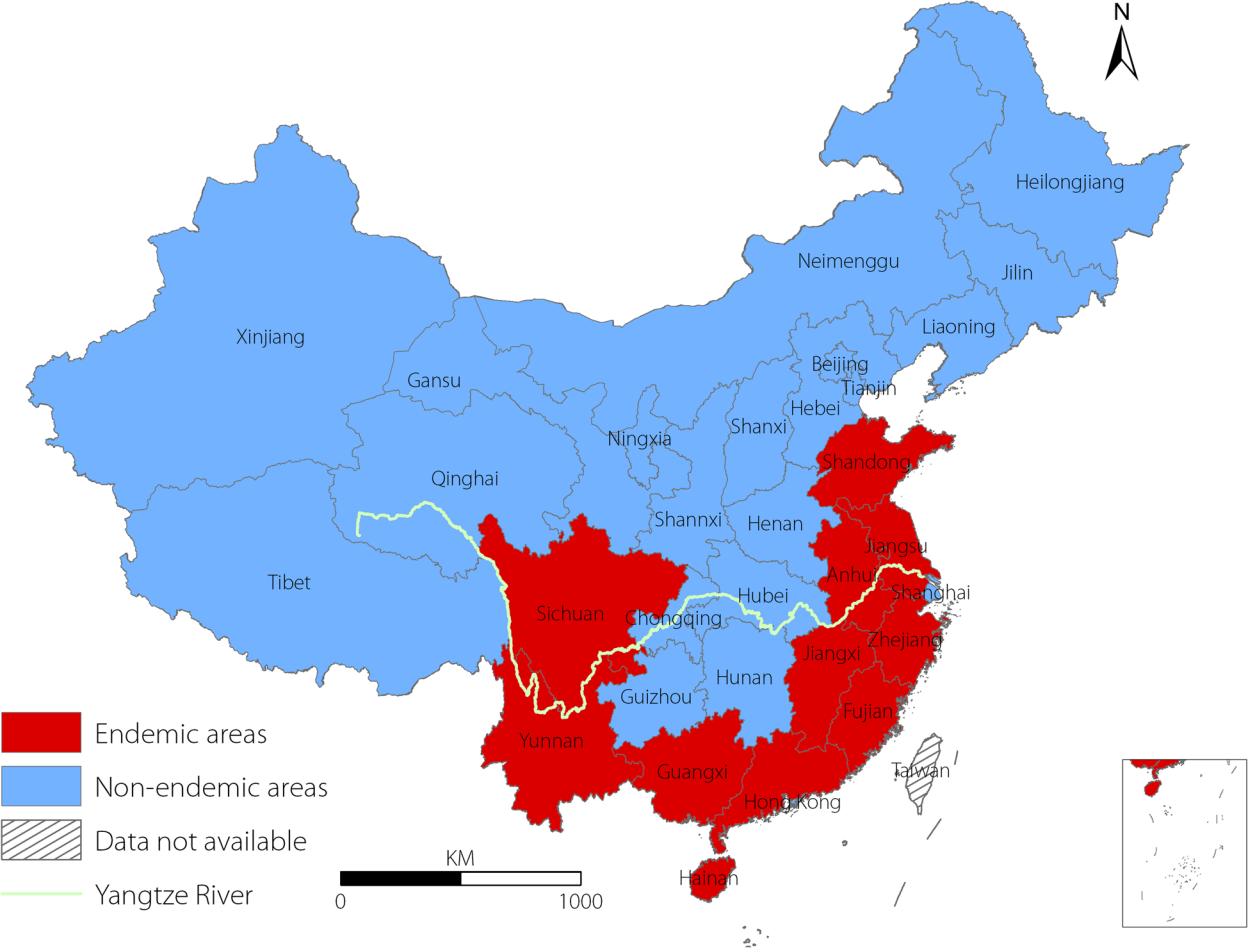
Geographical distribution of endemic and nonendemic areas for scrub typhus in China

We included probable and confirmed human cases with an illness onset between January 1, 2006, and December 31, 2016, in the analysis. The medical care institutions assessed were divided into city level hospitals (including city and province hospitals), county level hospitals and primary medical centres (including township hospitals and community health centres). City hospitals generally represent the highest medical care level in China, followed by county hospitals and primary medical centres. The proportion of probable and laboratory-confirmed cases from 2006 to 2016 was calculated and summarized overall and according to the three levels of medical institutions. We created a contrast bar to explore the relationship between the proportion of laboratory-confirmed cases and the number of cases reported by each province. To analyse the spatial distribution of laboratory-confirmed cases, we created geographical maps of the proportion of laboratory-confirmed cases overall as well as according to the three levels of medical care institutions. The provinces in China were divided into endemic and nonendemic areas for scrub typhus based on published articles (Fig. 1) [21, 22].

The interval from illness onset to case diagnosis (T_diag_) during the years 2006–2016 was calculated and compared. A box figure was created to illustrate the difference in the T_diag_ for laboratory-confirmed and probable cases among the three levels of medical care institutions. In addition, we calculated the overall T_diag_ and the T_diag_ for the three levels of medical care institutions of the different provinces. The Mann-Whitney U test was applied to examine whether the T_diag_ was significantly different between fatal and nonfatal cases and between confirmed and probable cases both overall and according to the three levels of medical care institutions. The Jonckheere-Terpstra test was used to examine whether the T_diag_ decreased from 2006 to 2016 overall and according to the three levels of medical care institutions or increased with an increase in medical care level. In addition, the multivariate logistic regression model was employed to explore the relationship between the risk of death and the T_diag_, with a significance level of α = 0.05. Model factors included the T_diag_ (< 2 days, 2–7 days and ≥ 8 days), sex (female and male), age (< 40 years, 40–59 years, ≥ 60 years), residence (rural and urban), region (middle east, southeast, southwest and north and west) and occupation (farmer and nonfarmer).

*R* statistical software (version 3.4.1, R Foundation for Statistical Computing, Vienna, Austria) was utilized to generate graphs and perform statistical analyses, and ArcGIS software (version 10.2.2, ESRI, Redlands, CA, USA) was used to plot geographical patterns.

## Results

### Case diagnosis category

From 2006 to 2016, a total of 93 481 scrub typhus cases were recorded by 2527 hospitals throughout the country, of which 89 099 were probable cases (95.3%) and 4382 were confirmed cases (4.7%); 75 hospitals (including 11 primary medical centers, 30 county hospitals and 34 city hospitals) reported scrub typhus cases every year since 2006. Among the 2527 hospitals, 1103 (43.6%) were primary medical centers, but these hospitals reported only 18.2% of all cases; 915 (36.2%) were county hospitals and reported 53.1% of all cases; and 505 (20.0%) were city hospitals and reported 27.7% of all cases (Table 2).

**Table 2:** Primary characteristics of the diagnosis and reporting of scrub typhus cases in China, 2006–2016.

Characteristics	2006	2007	2008	2009	2010	2011	2012	2013	2014	2015	2016	Overall
Number of cases	1254	1340	2613	3237	4088	6020	8928	11 111	16 035	17 293	21 562	93 481
Number of probable cases (%)	1049 (83.7)^a^	1187 (88.6)	2360 (90.3)	2939 (90.8)	3811 (93.2)	5630 (93.5)	8555 (95.8)	10 536 (94.8)	15 339 (95.7)	16 696 (96.5)	20 997 (97.4)	89 099 (95.3)
Number of confirmed cases (%)	205 (16.3)	153 (11.4)	253 (9.7)	298 (9.2)	277 (6.8)	390 (6.5)	373 (4.2)	575 (5.2)	696 (4.3)	597 (3.5)	565 (2.6)	4382 (4.7)
Number of fatal cases	0	1	3	0	11	6	9	4	8	3	12	5
Mortality rate (per 100 000)	0	0.00008	0.00023	0	0.00082	0.00045	0.00066	0.00029	0.00058	0.00022	0.00087	0.0003
Number of cases by medical care institution level												
City hospitals (*n*, %)	324 (25.8)	315 (23.5)	688 (26.3)	853 (26.4)	1118 (27.3)	1527 (25.4)	2585 (29.0)	3244 (29.2)	4915 (30.7)	4838 (28.0)	5488 (25.5)	25 895 (27.7)
County hospitals (*n*, %)	688 (54.9)	749 (55.9)	1376 (52.7)	1671 (51.6)	2144 (52.4)	3249 (54.0)	4507 (50.5)	5495 (49.5)	8264 (51.5)	9574 (55.4)	11 966 (55.5)	49 683 (53.1)
Primary medical centres (*n*, %)	201 (16.0)	247 (18.4)	460 (17.6)	648 (20.0)	760 (18.6)	1141 (19.0)	1743 (19.5)	2331 (21.0)	2855 (17.8)	2834 (16.4)	3758 (17.4)	16 978 (18.2)
Number of medical care institutions reporting cases	229	291	433	455	523	749	906	1009	1134	1229	1474	2527
City hospitals (*n*, %)	73 (31.9)	78 (26.8)	117 (27.0)	135 (29.7)	147 (28.1)	180 (24.0)	219 (24.2)	260 (25.8)	287 (25.3)	290 (23.6)	318 (21.6)	505 (20.0)
County hospitals (*n*, %)	101 (44.1)	127 (43.6)	196 (45.3)	181 (39.8)	209 (40.0)	292 (39.0)	344 (38.0)	381 (37.8)	446 (39.3)	513 (41.7)	624 (42.3)	915 (36.2)
Primary medical centres (*n*, %)	55 (24.0)	85 (29.2)	119 (27.5)	137 (30.1)	165 (31.5)	276 (36.8)	342 (37.7)	367 (36.4)	400 (35.3)	424 (34.5)	531 (36.0)	1103 (43.6)
Interval from illness onset to diagnosis (median, IQR)	7 (3–11)^b^	7 (3–10)	7 (3–10)	6 (2–10)	6 (2–9)	5 (2–9)	5 (2–9)	5 (2–9)	5 (2–9)	5 (2–8)	5 (2–8)	5 (2–9)
City hospitals (median, IQR)	8 (4–14)	8 (4–12)	8 (3–14)	7 (2–12)	7 (3–13)	7 (2–12)	6 (2–11)	7 (2–11)	6 (2–11)	6 (1–11)	6 (1–10)	6 (2–11)
County hospitals (median, IQR)	7 (3–11)	7 (3–10)	7 (3–10)	6 (2–9)	6 (3–9)	6 (2–9)	5 (2–8)	5 (2–8)	5 (2–8)	5 (3–8)	5 (2–8)	5 (2–8)
Primary medical centres (median, IQR)	6 (3–8.5)	5 (2–8)	4 (1–8)	5 (2–8)	3 (1–7)	4 (1–6)	4 (1–6)	4 (1–6)	4 (1–7)	3 (1–6)	3 (1–6)	4 (1–7)

^a^The data are presented as the number(%) of cases, unless otherwise indicated. ^b^*IQR* Interquartile range (P25 − P75).

**Fig. 2:**
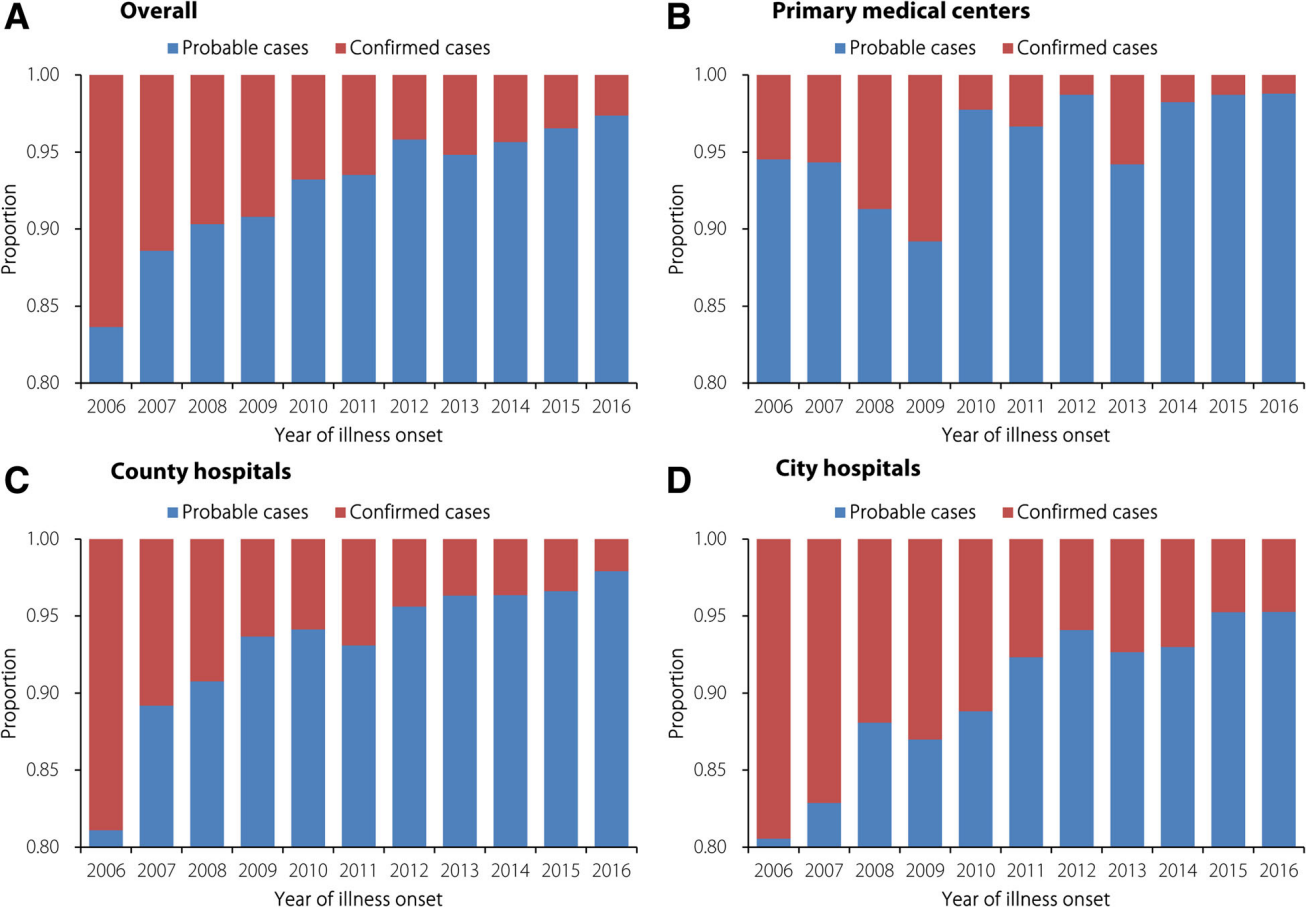
Proportion of probable and confirmed cases reported by year; **a** overall; **b** primary medical centers; **c** county hospitals; **d** city hospitals

**Fig. 3:**
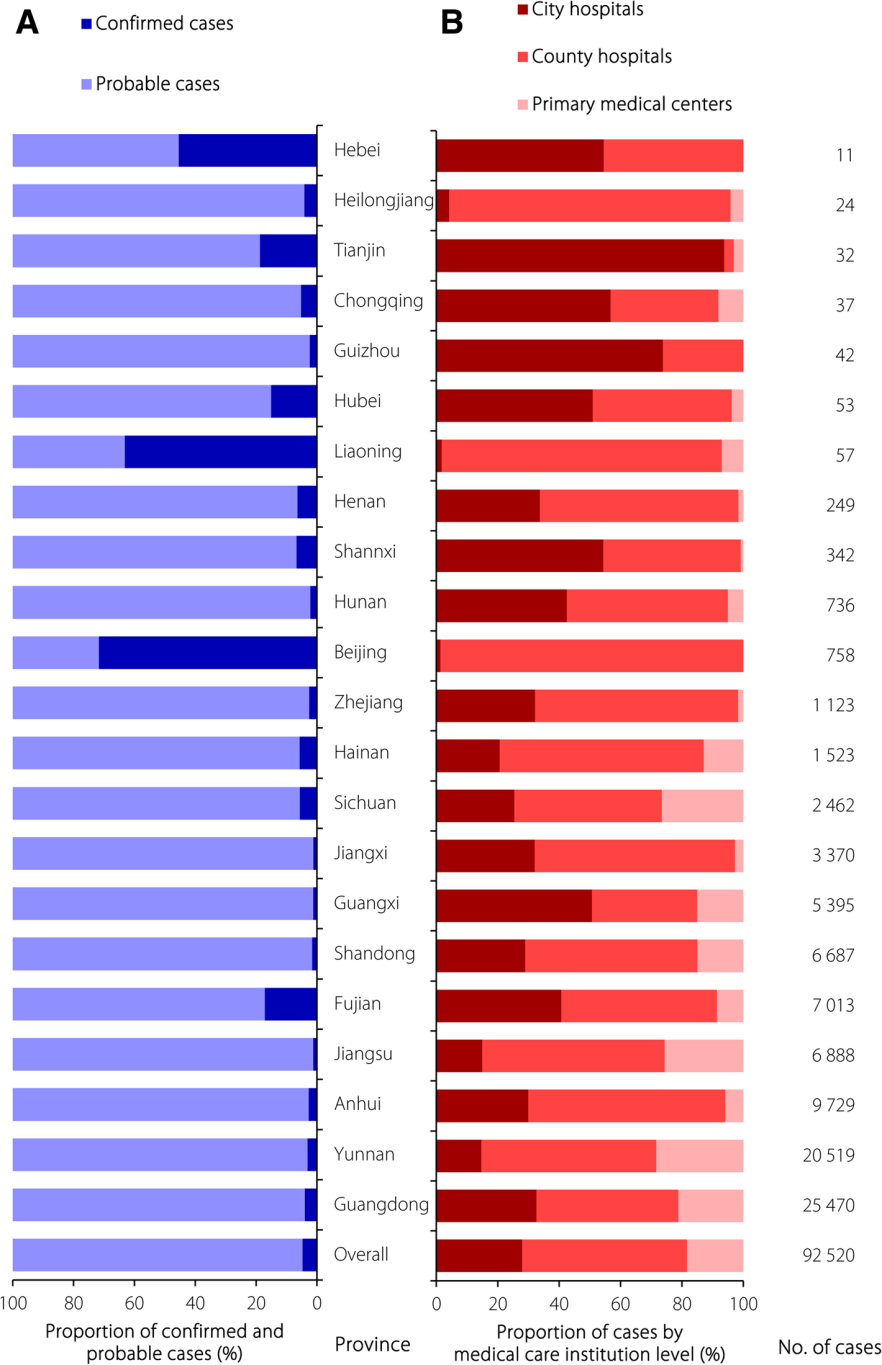
Proportion of cases reported by category and proportion of cases reported according to the three levels of medical care institutions and provinces for scrub typhus in China, 2006–2016; **a** proportion of probable and confirmed cases reported by province; **b** proportion of cases reported according to the three levels of medical care institutions and provinces

**Fig. 4:**
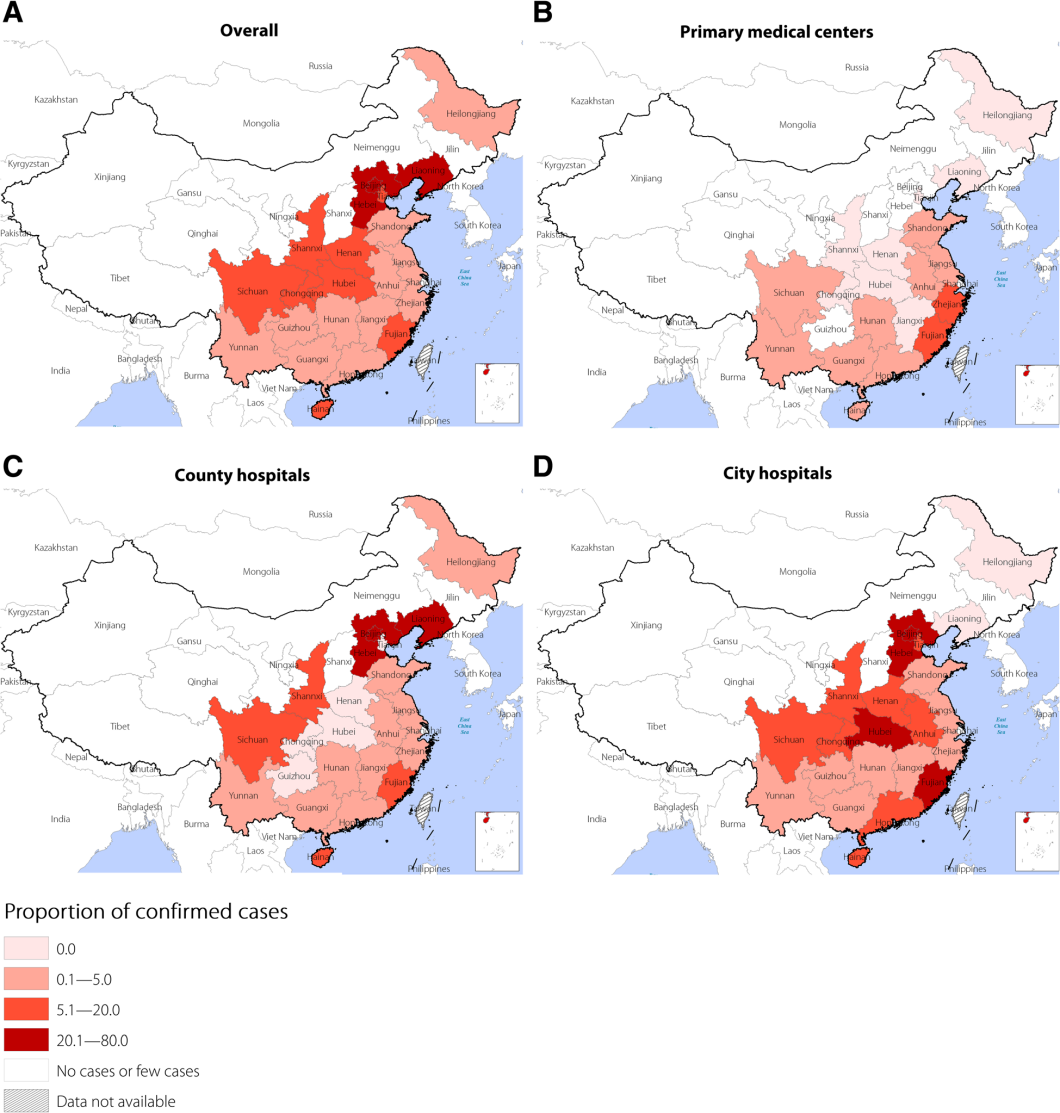
Proportion of confirmed cases reported according to the different levels of medical care institutions and provinces; **a** overall proportion of confirmed cases by province; **b** proportion of confirmed cases reported according to the primary medical centers and provinces; **c** proportion of confirmed cases reported by county hospitals and provinces; **d** proportion of confirmed cases reported by city hospitals per province

The overall proportion of confirmed cases was 4.7%, varying greatly among the primary medical centres (2.8%), county hospitals (4.2%), and city hospitals (6.3%) (Table 2). Notably, the proportion of laboratory-confirmed cases constantly decreased from 16.3% in 2006 to 2.6% in 2016, and the same decreasing trend was found among all three levels of medical care institutions (Fig. 2). A total of 91 099 (97.4%) cases in Guangdong, Yunnan and the other 11 provinces constituting the main epidemic areas in China were reported; the average proportion of confirmed cases among these provinces was only 4.1%. However, 2345 (2.5%) cases in Beijing, Hunan and the other 11 provinces constituting the areas of China not traditionally considered epidemic areas were reported, and the average proportion of confirmed cases among these provinces was 28.1% (Fig. 3). Additionally, there was a high proportion of confirmed cases reported by primary medical centres in the southeast coastal and southwest border areas of China, such as Fujian, Zhejiang and Yunnan provinces, which are in the main epidemic area for scrub typhus in China. In contrast, county and city hospitals in inland provinces/municipality such as Beijing, Hebei, Liaoning and Hubei, which are in an area of China not traditionally considered an epidemic area for scrub typhus, reported a high proportion of confirmed cases (Fig. 4).

### Case diagnosis time

**Fig. 5:**
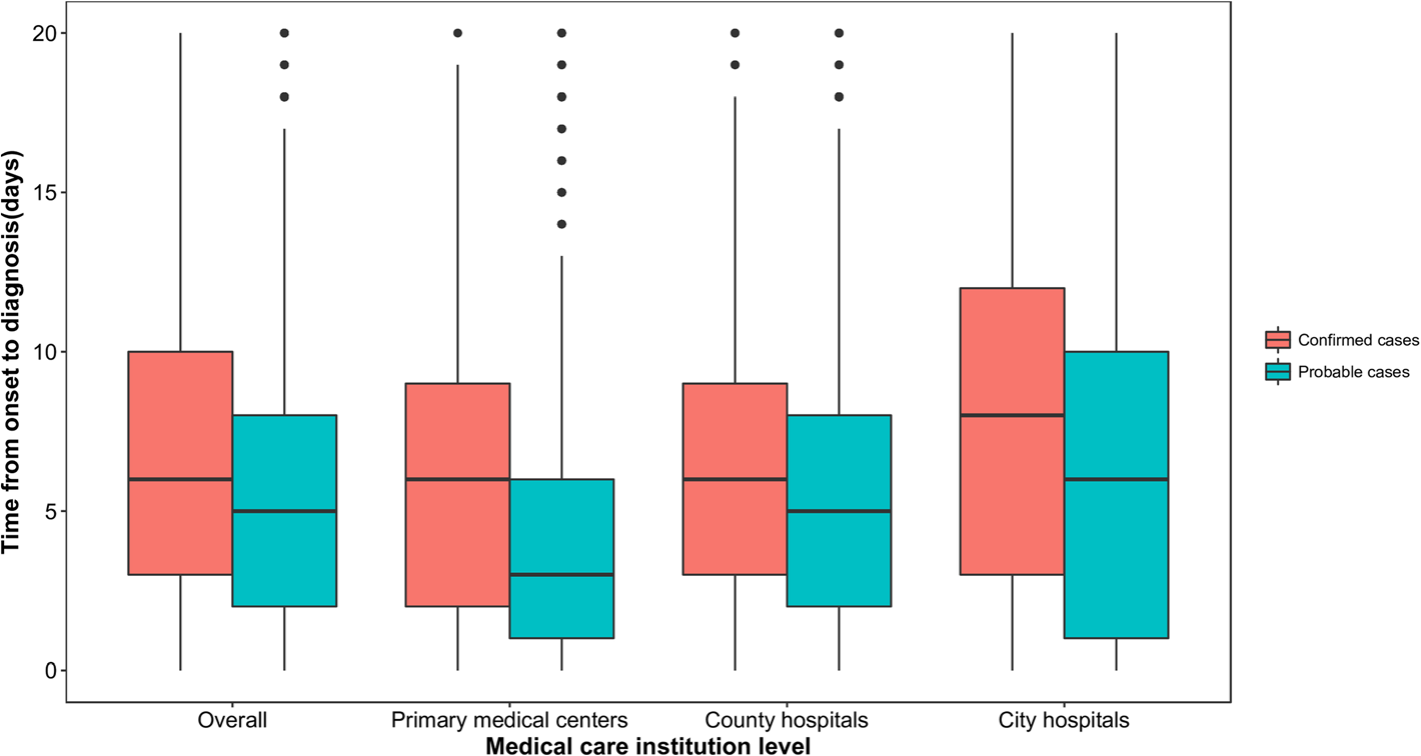
Time from onset to diagnosis for probable (blue bars) and confirmed (red bars) cases by medical care institution level

During the combined years of 2006 to 2016, the median T_diag_ was five days (interquartile range [IQR]: 2–9), with most cases (68.1%) diagnosed within one week of symptom onset and 91.2% of cases diagnosed within two weeks. The confirmed cases (median: 7, IQR: 3–12) had a longer T_diag_ than did the probable cases (median: 5, IQR: 2–9 days) (*Z* = 21.702, *P* < 0.01). The median T_diag_ decreased from seven days (IQR: 3–11 days) in 2006 to five days (IQR: 2–8 days) in 2016 (*Z* = 18.150, *P* < 0.01), and the same decreasing trend was found for all three levels of medical care institutions (Table 2). Additionally, the T_diag_ for both confirmed and probable cases increased with increasing medical care institution level (confirmed cases: *χ*^*2*^ = 10.720, *P* < 0.01; probable cases: *χ*^*2*^ = 42.920, *P* < 0.01), and the confirmed cases had a longer T_diag_ than did the probable cases for all three levels of medical care institutions (primary medical centers: *Z* = 10.394, *P* < 0.01; county hospitals: *Z* = 9.378, *P* < 0.01; city hospitals: *Z* = 14.339, *P* < 0.01) (Fig. 5). Furthermore, cases reported in areas not traditionally considered epidemic areas (inland provinces) had a T_diag_ longer than that of cases reported in the main epidemic area (Chinese southeast coastal and southwest border provinces) for all three levels of medical care institutions from 22 provinces in China from 2006 to 2016 (Table 3).

**Table 3:** Interval time from illness onset to diagnosis of scrub typhus cases

Province/ municipality/ autonomous region	Interval between illness onset and case diagnosis, days (median [IQR])				Deaths(*n* = 57)
	Overall	Primary medical centre	County hospital	City hospital	
Guangdong (*n* = 25 586)	5 (1–9)	4 (1–8)	5 (2–9)	5 (1–10)	28
Yunnan (*n* = 20 690)	4 (2–7)	3 (1–5)	5 (2–8)	7 (4–11)	11
Anhui (*N* = 9737)	5 (2–8)	1 (1–2)	5 (2–7)	6 (2–10)	1
Jiangsu (*n* = 7127)	5 (2–9)	5 (3–7)	5 (2–9)	7 (2–11)	0
Fujian (*n* = 7062)	6 (2–10)	5 (2–9)	6 (2–10)	6 (0–11)	7
Shandong (*n* = 6866)	6 (4–9)	5 (3–7)	6 (4–9)	7 (4–10)	1
Guangxi (*n* = 5476)	6 (2–10)	4 (1–7)	5 (2–9)	7 (3–11)	5
Jiangxi (*n* = 3371)	8 (4–13)	5 (2–8)	7 (4–11)	11 (6–17)	1
Sichuan (*n* = 2508)	6 (3–10)	4 (1–6)	6 (3–9)	10 (6–15.25)	0
Hainan (*n* = 1541)	5 (2–9)	4 (1–6)	5 (2–8)	8 (4–13)	0
Zhejiang (*n* = 1135)	5 (0–9)	5 (1–8)	5 (2–9)	1 (0–9)	0
Beijing (*n* = 759)	5.5 (3.75–8)	-^a^	5 (3.25–7)	12.5 (3.75–19)	0
Hunan (*n* = 738)	6 (3–11)	2 (0–4)	5 (2–8)	10 (5–16)	1
Shanxi (*n* = 342)	6 (4–9)	–	5 (4–7)	7 (4–11)	0
Henan (*n* = 249)	6 (2–9)	7.5 (2.5–9.5)	5 (2–8)	8 (4–12)	0
Liaoning (*n* = 57)	9 (7–14)	8 (6.5–12.5)	10 (7–14)	2 (2–2)	0
Hubei (*n* = 53)	5 (1.5–9)	–	4 (1.25–8)	7 (0–11)	1
Guizhou (*n* = 42)	7 (3–13.25)	–	4 (3–7)	8 (4–14)	1
Chongqing (*n* = 38)	3 (0–9)	–	5 (0.5–10)	2 (0–9)	0
Tianjin (*n* = 32)	5 (2.25–8.75)	–	11 (11–11)	5 (1.75–8.25)	0
Heilongjiang (*n* = 24)	6.5 (5–8.75)	–	7 (5–9)	0 (0–0)	0
Hebei (*n* = 11)	8 (3–11)	–	8 (5–9.5)	7.5 (0–25.5)	0
Overall	5 (2–7)	4 (1–7)	5 (2–8)	6 (2–11)	57

^a^ “-” indicates no data or few data (less than ten cases).

### Diagnosis of mortality cases

A total of 57 deaths were recorded throughout the country during the 11 years of this study, and the mean annual mortality rate was 0.0003 per 100 000. The median interval time between illness onset and death was 8 days (IQR: 3–10 days). The median interval from illness onset to case diagnosis in fatal cases (median: 7 days, IQR: 3–11 days) was significantly higher than that in nonfatal cases (median: 5 days, IQR: 2–9 days) (*χ*^2^ = 4.013, *P* < 0.05). Multivariate logistic regression analysis showed that cases with a T_diag_ of > 7 days had a risk of death that was 2.2 times higher (*OR* = 2.21 [95% *CI*: 1.05–5.21]) than that of cases with a T_diag_ of < 2 days.

## Discussion

By analysing a surveillance dataset spanning 11 years, we found that the interval from illness onset to diagnosis of scrub typhus cases decreased greatly. However, the majority of scrub typhus cases were probable cases, and the proportion of confirmed cases decreased sharply during the study period. The same decreasing trend was found for all three levels of medical care institutions. Moreover, late diagnosis of scrub typhus increased the risk of death.

The T_diag_ decreased from seven days in 2006 to five days in 2016, which was mainly attributed to the development of medical standards, including quality promotion by medical service staff, medical equipment improvement and management-level upgrades. Additionally, China has made enormous strides in improving health in the past few decades [23], with a significant improvement in health awareness that would shorten the time from illness onset to seeking medical service, which would decrease the T_diag_. In our study, probable cases had a lower T_diag_ than did confirmed cases, and the proportion of probable cases increased since 2006, indicating that the decrease in the T_diag_ during the 2006–2016 period was partially due to the increasing proportion of probable cases.

In this study, 95.3% of the scrub typhus cases reported by Chinese medical care institutions were probable cases. Thus, according to the case definition, the majority of cases presented with eschars/ulcers. However, a wide range of eschar/ulcer occurrence (7–97%) has been found in other studies, and according to the prevention guideline published by the China CDC in 2009, only approximately 50% of scrub typhus cases develop the symptom of eschars/ulcers. These findings suggest that some cases might be misdiagnosed or underreported [5, 24].

Southeast coastal and southwest border provinces are the major epidemic areas for scrub typhus in China, reporting over 90% of all cases in the country [21, 22]. Doctors in these provinces have greater experience in diagnosing and treating scrub typhus patients than do doctors in areas not traditionally considered epidemic areas, and thus scrub typhus cases are diagnosed relatively quickly in those epidemic areas. Because of the low awareness of scrub typhus in basic medical care institutions in areas not traditionally considered epidemic areas, most cases in these areas are referred to county and city hospitals for confirmatory testing, which may prolong the interval from illness onset to case diagnosis. This occurrence also explains the low proportion of confirmed cases in primary medical centres in subepidemic areas. Additionally, in an area not traditionally considered an epidemic area, the lack of experience leads to more laboratory tests being performed before diagnosis, which thus increases the proportion of confirmed scrub typhus cases in these areas.

As scrub typhus is treatable with specific antimicrobial medicines, early and accurate diagnosis is essential for reducing the risk of severe complications and death. Our study highlights the need for research and development of inexpensive, accurate point-of-care diagnostic tests for the acute phase of the infection in the clinical setting. The IFA is currently the gold standard reference diagnostic method recommended by the WHO [25]. However, there is a lack of standardization and lab-to-lab variability in cutoff titers among reported results [9, 26]. Moreover, the majority of seroepidemiology studies have utilized a single cutoff titer to determine positive results, with a wide range of reported cutoff values from 1:10 to 1:400, leading to subjective endpoints that cause incomparable results [9, 26]. In addition, the high cost and requirement of experienced personnel to evaluate IFA slides have limited its use in resource-restricted areas. The Weil-Felix agglutination test, which is widely used in each level of Chinese medical institutions as the laboratory diagnostic method for scrub typhus, is an inexpensive option for diagnosis of rickettsial infections, but it has poor sensitivity and specificity [3, 27–29]. Real-time PCR assays such as the 47-kDa, 56-kDa, and GroEL gene assays, which detect 10–50 copies/μl of *Ot*, are increasingly being adopted. Indeed, employing type-specific genes (e.g., 56 kDa and 47 kDa for *Ot*) increases the specificity of real-time PCR when genus-specific genes are used (e.g., 17 kDa genes for *Rickettsia* spp.) [3]. Regardless, real-time PCR assays are limited because the best specimens for detection are from eschar biopsies, followed by the buffy coat, whole blood and blood clots, and obtaining eschar biopsies is challenging [3, 28, 30–32]. Additionally, the high cost of equipment and expertise of the operator make PCR assays impractical in primary medical centres and county hospitals. ELISAs and ELISA variants, such as in commercially available dipstick tests, use either pooled cell lysates of different *O. tsutsugamushi* strains, recombinant p56 or other outer membrane proteins as the antigen. ELISAs provide sensitive and specific test results and may eventually replace the IFA. For example, the sensitivity and specificity in a study involving Thailand patients were 97 and 89% for IgG (1:1600 cutoff) and 94 and 91% for IgM (1:400 cutoff), respectively [9, 33]. Additionally, these commercially available assays are easy to use and can be performed in the primary medical care setting [33, 34]. Blacksell et al. reported experience with the InBios Scrub Typhus Detect IgM ELISA, which has been shown to be easy to use and affordable and to have adequate accuracy for screening and diagnosis [29]. Furthermore, immunochromatographic rapid diagnostic tests (RDTs or ICTs) have been developed in the last decade based on the need for tools to rapidly diagnose scrub typhus, and the ability to produce high-quality recombinant protein antigens has enabled development of more specific and sensitive RDTs. Recently, a second-generation lateral-flow format product, Scrub Typhus Detect (ST Detect), manufactured by InBios (Seattle, WA, USA), using a mixture of 4 recombinant 56-kDa antigens showed sensitivity for IgM detection of 99.25 and 100% among Indian and Thai patients, respectively [35, 36]. RDTs produce results rapidly and follow a simple protocol with no need for sophisticated electrical equipment, meeting the requirements of a field-deployable, point-of-care diagnostic assay for early diagnosis of scrub typhus with military relevance. Additionally, RDTs are attractive for use in rural areas where diagnostics such as ELISA and IFA may not be available [9].

Our study showed an increase in reported scrub typhus cases since 2006, which is similar to a previous report on scrub typhus in China from 2006 to 2014 [37], and 17 293 and 21 562 cases were reported in 2015 and 2016, respectively. The substantial increase in reported cases is a public health concern, and the factors driving this increase need to be explored through further field study and assessment.

Our study has some limitations. First, all data used were collected from a passive disease surveillance system, and the data quality may be influenced by the completeness and accuracy of the data over the studied time period. Thus, there may be even more cases than currently reported. More cases might be underreported in primary medical centres than in county or city hospitals, which would affect the results of this study. Moreover, only the diagnosis of scrub typhus was evaluated in this study; other aspects, such as the completeness and validity of the data and the sensitivity and specificity of the scrub typhus surveillance system, need to be further assessed in the future. Nonetheless, the data used in this study are currently the most comprehensive and reliable data on scrub typhus at the national and subnational levels in China, and all results from these nationwide data were carefully analysed and interpreted to reduce confounding factors.

## Conclusions

Our study showed that the interval from illness onset to diagnosis of scrub typhus cases decreased greatly; however, the proportion of cases diagnosed with laboratory-confirmed results was low among the different levels of medical care institutions. Our study suggests that more efforts should be made to develop an inexpensive and accurate point-of-care diagnostic test for the acute phase of the infection to reduce both the risk of death and the misuse of antibiotics associated with scrub typhus. Additionally, more training concerning scrub typhus diagnosis should be developed to enhance awareness among clinicians in endemic areas of China. Furthermore, there should be more discussion on the current case definition criteria and case category in the national guideline for scrub typhus.

## Additional files

Additional file 1: Multilingual abstracts in the five official working languages of the United Nations. (PDF 383 kb)

Additional file 2: Demographic characteristics, including date of illness onset, date of diagnosis, date of death, and medical care institution. (XLSX 4541 kb)

## Data Availability

All data generated or analysed during this study are included in this published article and its supplementary information files.

## Abbreviations

ELISA: Enzyme-linked immunosorbent assay
IFA: Indirect immunofluorescence antibody assay
IQR: Interquartile range
NNIDRIS: National Notifiable Infectious Disease Reporting Information System
OR: Odds ratio
*Ot*: *Orientia tsutsugamushi*
PCR: Polymerase chain reaction
RDTs or ICTs: Immunochromatographic rapid diagnostic tests
SARS: Severe acute respiratory syndrome
Tdiag: The interval from illness onset to case diagnosis
WHO: World Health Organization
