# Supplementary material for: Evaluation of scrub typhus diagnosis in China: analysis of nationwide surveillance data from 2006 to 2016
Source: Infect Dis Poverty. 2019 Jun 29;8:59. doi: 10.1186/s40249-019-0566-0 (PMC6599364; doi:10.1186/s40249-019-0566-0)
Supplement: Supplementary file 1 — Multilingual abstracts in the five official working languages of the United Nations. (PDF 383 kb) [file 40249_2019_566_MOESM1_ESM.pdf]

Translation of the abstract into the five official working languages of the United Nations

تقييم تشخيص التيفوئيد في المقشر الصين: تحليل بيانات المراقبة علي الصعيد الوطني من 2006 إلى 2016

Hua-Lei Xin, Jian-Xing Yu, Mao-Gui Hu, Fa-Chun Jiang, Xiao-Jing Li, Li-Ping Wang, Ji-Lei Huang, Jin-Feng Wang, Jun-Ling Sun and Zhong-Jie Li

مجرده

الخلفية: فرك التيفوئيد هو مرض يهدد الحياة بسبب اورينتيا تسوتسوغامشي ، والادويه المضادة للميكروبات المحددة المتاحة. التشخيص المبكر والدقيق ضروري للحد من خطر المضاعفات الشديدة والوفاة. وفي هذه الدراسة ، استهدفنا تقييم حاله تشخيص الحالة بين مؤسسات الرعاية الطبية والمناطق الجغرافية في الصين ، ستفيد النتائج كلا من الممارسة السريرية ونظام مراقبه الامراض.

الطرق: استخرجنا بيانات حاله الفرك الفردية لعامي 2006 – 2016 من نظام وطني لمراقبه الامراض في الصين. وقورنت فئة التشخيص وفتره الفاصل الزمني من بداية المرض إلى التشخيص بين ثلاثه مستويات من مؤسسات الرعاية الطبية والمقاطعات. وقد أجريت طريقه التحليل الوصفي في دراستنا.

النتائج: خلال فتره الدراسة التي بلغت 11 عاما ، سجلت 93 481 حاله من حالات التيفوئيد ، بما في ذلك 57 وفاه ، في نظام المراقبة علي الصعيد الوطني. وكانت النسبة الاجماليه للحالات المؤكدة مختبريا 4.7 في المائة فقط ، وتفاوتت هذه النسبة تفاوتا كبيرا بين المراكز الطبية الاوليه (2.8 في المائة) ، ومستشفيات علي مستوي المقاطعات (4.2 في المائة) ، ومستشفيات علي مستوي المدن (6.3 في المائة). ومن الجدير بالذكر ان نسبه الحالات المؤكدة مختبريا قد انخفضت باستمرار من 16.3 في المائة في 2006 إلى 2.6 في المائة في 2016 ، ووجد نفس الاتجاه المتناقص بين جميع المستويات الثلاثة لمؤسسات الرعاية الطبية. كان الفاصل الزمني من بداية المرض إلى تشخيص الحالة (Tdiag) لجميع الحالات 5 أيام (المدى الربع [IQR]: 2 – 9 أيام) وانخفض من 7 أيام (IQR: 3 – 11 يوما) في 2006 إلى 5 أيام (IQR: 2 – 8 أيام) في 2016. وكان خطر الوفاة بالنسبة للمرضي مع Tdiag من > 7 أيام اعلي 2.2 (أو = 2.21 ، 95 ٪ CI: 1.05 – 5-12) من المرضي الذين يعانون من Tdiag من < 2 يوما.

الاستنتاجات: انخفض الوقت الفاصل من الزمني من بداية المرض إلى التشخيص لحالات التيفوئيد فرك بشكل كبير في الصين. ومع ذلك ، يجب زيادة معدل تشخيص الحالات مع النتائج المؤكدة مختبريا بين جميع مستويات مؤسسات الرعاية الطبية للحد من خطر الوفاة وسوء استخدام المضادات الحيوية المرتبطة بالتيفوئيد فرك

Translated from English version into Arabic by Hamza Faleh Hamza,

中国恙虫病诊断情况评估：来自 2006-2016 年全国监测数据的分析

Hua-Lei Xin, Jian-Xing Yu, Mao-Gui Hu, Fa-Chun Jiang, Xiao-Jing Li, Li-Ping Wang, Ji-Lei Huang, Jin-Feng Wang, Jun-Ling Sun and Zhong-Jie Li

摘要：

背景：恙虫病是一种由恙虫病东方体引起，需要特殊抗菌药物治疗的可威胁生命的疾病，早期准确诊断对减少并发症和死亡非常重要。本次研究的主要目的是对中国不同医疗卫生机构、不同地域的恙虫病诊断情况进行评估，以促进中国恙虫病诊疗行为和监测系统改善。

方法：我们从中国疾病监测信息系统中提取 2006-2016 年恙虫病个案数据，对三个级别医疗机构的恙虫病诊断类别和发病到诊断时间进行比较。本研究应用的是描述性流行病学方法。

结果：2006-2016 年中国疾病监测信息系统共报告 93 481 例恙虫病病例，其中 57 例死亡。实验室确诊病例只占 4.7%，且实验室确诊病例比例在各级医疗机构存在差异，在初级医疗机构中占 2.8%，在县级医疗机构中占 4.2%，在市级医疗机构中占 6.3%。值得注意的是实验

室确诊病例比例由 2006 年的 16.3%下降至 2016 年的 2.6%，且这种下降趋势在三个级别医疗机构都出现。所有病例发病到诊断时间的中位数是 5 天（四分位间距：2-9 天），发病到诊断时间中位数由 2006 年的 7 天（四分位间距：3-11 天）下降至 2016 年的 5 天（四分位间距：2-8 天）。发病到诊断时间>7 天的病例死亡风险是<2 天的 2.2 倍（OR = 2.21, 95% CI: 1.05− 5.21）。

结论：中国恙虫病病例从发病到诊断的时间有较大改善，但各级医疗机构恙虫病实验室确诊病例的比例有待提高，以减少因误诊而导致的抗生素滥用和恙虫病所致的死亡。

Translated from English version into Chinese by Hua-Lei Xin

Évaluation du diagnostic du typhus tropical en Chine : analyse des données de surveillance nationales de 2006 à 2016

Hua-Lei Xin, Jian-Xing Yu, Mao-Gui Hu, Fa-Chun Jiang, Xiao-Jing Li, Li-Ping Wang, Ji-Lei Huang, Jin-Feng Wang, Jun-Ling Sun et Zhong-Jie Li

Résumé

Contexte : le typhus tropical, ou fièvre fluviale du Japon, est une maladie potentiellement mortelle causée par *Orientia tsutsugamushi*, contre laquelle il existe des médicaments antimicrobiens spécifiques. Un diagnostic précoce et précis est crucial pour réduire le risque de complications graves et de décès. Dans la présente étude, nous avons dressé le bilan de son diagnostic dans les établissements de soins médicaux et les régions géographiques de Chine. Les résultats de cette étude intéressent aussi bien la pratique clinique que le système de veille épidémiologique.

Méthodes : nous avons extrait les données des cas individuels de typhus tropical de 2006 à 2016 d'un système national de veille épidémiologique chinois. La catégorie de diagnostic et le délai entre l'apparition de la maladie et son diagnostic ont été comparés à trois niveaux d'établissements de santé et des provinces, en suivant une méthode d'analyse descriptive.

Résultats : sur la période de 11 ans étudiée, 93 481 cas de typhus tropical, dont 57 mortels, ont été enregistrés dans le système national de surveillance. La proportion totale de cas confirmés en laboratoire était seulement de 4,7 % et s'est avérée très variable entre les centres médicaux primaires (2,8 %), les hôpitaux de circonscription (4,2 %) et les hôpitaux urbains (6,3 %). On note en particulier que la proportion de cas confirmés en laboratoire a constamment baissé, de 16,3 % en 2006 à 2,6 % en 2016, et cette tendance à la baisse se retrouve aux trois niveaux des établissements médicaux. L'intervalle moyen entre l'apparition de la maladie et le diagnostic du cas (T_{diag}) était de 5 jours (fourchette interquartile [IQR] de 2 à 9 jours). Il a diminué de 7 jours (IQR de 3 à 11 jours) en 2006 à 5 jours (IQR de 2 à 8 jours) en 2016. Le risque de décès des patients était 2,2 fois plus élevé si T_{diag} > 7 jours (OR = 2,21, *IC* à 95 %de 1,05 à 5,21) que si T_{diag} < 2 jours.

Conclusions : le délai entre l'apparition de la maladie et le diagnostic du typhus tropical a beaucoup diminué en Chine. Le taux de diagnostic des cas confirmés en laboratoire doit cependant être augmenté à tous les niveaux des établissements de soins de santé, afin de réduire aussi bien le risque de décès que le mauvais usage des antibiotiques visant le typhus tropical.

Translated from English version into French by Suzanne Assenat, Revised by Eric Ragu, through

Оценка результатов диагностики цуцугамуши (японская лихорадка) в Китае: анализ общенациональных данных эпиднадзора за период с 2006 по 2016 год.

ХуаЛэй Синь, Цзянь-синь Юй, Мао Гуй Ху, Фа-Чунь Цзян, Сяо-Цзинь Ли, Ли Пин Ван, Цзи Лэй Хуань, Цзинь-Фэнь Ван, Чжун Линь Сунь и Чжун Цзинь Ли.

Реферат

Общая информация: цуцугамуши - опасное для жизни заболевание, вызываемое бактерией *Orientia tsutsugamushi*, для лечения которого существуют специальные противомикробные препараты.Ранняя и правильная диагностика заболевания крайне важна для снижения риска тяжелых осложнений и смерти. Целью данного исследования является оценка ситуации диагностики заболеваний в медицинских учреждениях и различных географических регионах Китая, результаты которой принесут пользу как клинической практике, так и системе эпидемиологического надзора.

Методы: Мы собрали данные о случаях заболевания цуцугамуши у отдельных пациентов за 2006-2016 годы из национальной системы эпидемиологического надзора Китая. Категория диагноза и интервал времени от начала заболевания до постановки диагноза были сопоставлены между тремя типами медицинских учреждений и провинциями Китая. В нашем исследовании использовался описательный метод анализа.

Результаты:За 11-летний период исследования в общенациональной системе наблюдения было зарегистрировано 93 481 случаев заболевания цуцугамуши, в том числе 57 с летальным исходом. Общая доля лабораторно подтвержденных случаев составила лишь 4,7 %, и эта доля значительно варьировалась между центрами первичной медицинской помощи (2,8 %), больницами сельского уровня (4,2 %) и больницами городского уровня (6,3 %). Примечательно, что доля лабораторно подтвержденных случаев заболевания последовательно снизилась с 16,3 % в 2006 году до 2,6 % в 2016 году, такая же тенденция наблюдается во всех трех типах лечебных учреждений. Интервал от начала заболевания до постановки диагноза ($Т_{диаг}$) для всех случаев составлял 5 дней (межквартильный размах [МКР]: 2-9 дней) и уменьшился с 7 дней (МКР: 3-11 дней) в 2006 году до 5 дней (МКР: 2-8 дней) в 2016 году. Риск смертности среди пациентов с интервалом $Т_{диаг} > 7$ дней был в 2,2 раза выше (отношение шансов = 2,21, 95 % *ДИ*: 1,05-5,21), чем у пациентов с интервалом $Т_{диаг} < 2$ дней.

Выводы: Интервал времени от начала заболевания до постановки диагноза цуцугамуши значительно сократился в Китае; однако, для снижения риска смертности и злоупотребления антибиотиками, связанными с лихорадкой, уровень диагностики лабораторно подтвержденных случаев должен быть улучшен для медицинских учреждений всех типов.

Translated from English version into Russian by Dina Martin-Bouyer, Revised by Michael Orlov,

through

Evaluación del diagnóstico del tifus de los matorrales en China: análisis de los datos de vigilancia a nivel nacional entre 2006 y 2016

Hua-Lei Xin, Jian-Xing Yu, Mao-Gui Hu, Fa-Chun Jiang, Xiao-Jing Li, Li-Ping Wang, Ji-Lei Huang, Jin-Feng Wang, Jun-Ling Sun y Zhong-Jie Li

Resumen

Antecedentes: El tifus de los matorrales es una enfermedad potencialmente mortal causada por la *Orientia tsutsugamushi*, y hay medicina antimicrobiana específica disponible. El diagnóstico precoz y preciso es esencial para reducir el riesgo de complicaciones graves y de muerte. El objetivo de nuestro estudio fue evaluar la situación diagnóstica de los casos en las instituciones médicas y en las regiones geográficas de China, y los resultados beneficiarán tanto la práctica clínica como el sistema de vigilancia de enfermedades.

Métodos: Recolectamos los datos de los casos individuales de tifus de los matorrales entre 2006 y 2016 del sistema nacional de vigilancia de enfermedades de China. Comparamos la categoría del diagnóstico y el intervalo de tiempo desde el inicio de la enfermedad hasta el diagnóstico entre instituciones de los tres niveles de atención médica y a nivel provincial. En nuestro estudio se aplicó el método de análisis descriptivo.

Resultados: Durante los 11 años del estudio, se registraron 93.481 casos de tifus de los matorrales en el sistema nacional de vigilancia, de los cuales 57 fueron mortales. La proporción total de casos confirmados por laboratorio fue tan solo del 4,7% y varió considerablemente entre los centros de atención primaria (2,8 %), los hospitales del nivel provincial (4,2 %) y los hospitales del nivel de ciudad (6,3 %). Es de destacar que la proporción de casos confirmados por laboratorio ha disminuido sistemáticamente del 16,3 % en 2006 al 2,6 % en 2016, observándose la misma tendencia decreciente en los tres niveles de atención médica. El intervalo de tiempo desde el inicio de la enfermedad hasta el diagnóstico ($T_{diag.}$) de todos los casos fue de 5 días (rango intercuartil [RIQ]: 2-9 días) y disminuyó de 7 días (RIQ: 3-11 días) en 2006 a 5 días (RIQ: 2-8 días) en 2016. El riesgo de muerte en pacientes con un $T_{diag.} > 7$ días fue 2,2 veces mayor (OR = 2,21; 95 % *IC*: 1,05-5,21) que en los pacientes con un $T_{diag.} < 2$ días.

Conclusiones: El intervalo desde el inicio de la enfermedad hasta el diagnóstico en los casos de tifus de los matorrales se ha reducido notablemente en China; no obstante, se debe mejorar la proporción de casos con diagnóstico confirmado por laboratorio en las instituciones de todos los niveles de atención médica para reducir tanto el riesgo de muerte como el uso indebido de antibióticos asociado con el tifus de los matorrales.

Translated from English version into Spanish by Carmen Blazquez, Revised by Maria Patricia, through

TRANSLATORS
WITHOUT BORDERS
